# Hospital at home enables safe and efficient outpatient management of intensively treated acute myeloid leukemia

**DOI:** 10.1007/s00520-026-11171-2

**Published:** 2026-09-10

**Authors:** Kristina Warolén, Ola Blennow, Anna Ravn Landtblom, Christer Nilsson, Linus Kullänger Schalling, Christine Andersson, Cecilia Lind, Sandra Hållander, Sara Westerberg, Ann-Sofie Stjernfeldt, Emma Sporre, Hillevi Leskinen, Linda Söderholm, Lovisa Wennström, Per Anders Broliden, Stefan Deneberg, Erik Espman, Lena von Bahr, Martin Jädersten

**Affiliations:** 1 Department of Hematology, Karolinska University Hospital, Stockholm, Sweden; 2 Division of Clinical Epidemiology, Department of Medicine Solna, Karolinska Institutet, Stockholm, Sweden; 3 Department of Infectious Diseases, Karolinska University Hospital, Stockholm, Sweden; 4 Center for Hematology and Regenerative Medicine, Department of Medicine, Karolinska Institutet, Stockholm, Sweden; 5 Capio Hospital at Home, Dalen Hospital, Stockholm, Sweden; 6 Section of Hematology and Coagulation, Sahlgrenska University Hospital, Gothenburg, Sweden; 7 Institute of Medicine, Sahlgrenska Academy, Gothenburg University, Gothenburg, Sweden

**Keywords:** Acute myeloid leukemia, Hematology, Home Care

## Abstract

**Purpose:**

Patients with acute myeloid leukemia (AML) spend a substantial amount of time in hospital while receiving intensive chemotherapy, affecting their quality of life and healthcare costs. This study evaluated the safety of outpatient management of febrile neutropenia with support from hospital at home (HaH).

**Method:**

Two cohorts of intensively treated patients with AML were retrospectively compared, one from Stockholm (*n* = 116) with access to HaH and another from Gothenburg (*n* = 49) without HaH support. Primary outcome was incidence of severe complications, defined as death or requirement of higher level of care.

**Results:**

During cycle 1 (C1), 109 of 116 patients (94%) in Stockholm were discharged, of whom 50 (46%) were readmitted. In Gothenburg, 48 of 49 patients (98%) remained in hospital for the entirety of the neutropenic phase. The incidence of severe complications, including fatal events and requirement of higher level of care, was comparable in the two cohorts. Out of 150 neutropenic episodes (during C1 and C2) among discharged Stockholm patients, febrile neutropenia developed in 130 cases (86.7%). Of these, 38 episodes (29%) were resolved with administration of antibiotics at home only.

**Conclusion:**

These findings demonstrate that outpatient management of AML patients treated with intensive chemotherapy is feasible and safe when supported by a structured HaH program in close collaboration with the hematology department. Implementation of at-home care has the potential to reduce the burden of prolonged periods of hospitalization for patients with AML and warrants further evaluation in prospective trials.

**Supplementary information:**

The online version contains supplementary material available at https://doi.org/10.1007/s00520-026-11171-2.

## Introduction

About 400 patients in Sweden are diagnosed with acute myeloid leukemia (AML) every year, and fit patients most often receive intensive chemotherapy (ICT), with or without subsequent allogeneic hematopoietic stem cell transplantation (HSCT). Novel non-intensive alternatives and targeted drugs have increased the treatment arsenal and improved outcome during the past decade. However, even in fit patients below the age of 70, more than a third succumb to the disease during the first year, and the 5-year overall survival is only around 40% (Swedish AML INCA register 2025).

Over the past decades, extensive efforts have been made in oncology to move patient care out from the hospital to the patient’s own home, where patients generally have better nutritional intake, are better mobilized, and have a better quality of life [1, 2]. In addition, inpatient stay is a major driver of healthcare costs [3–5]. AML patients receiving ICT are typically hospitalized for around 4 weeks during the first induction cycle and for 2 to 3 weeks during each consolidation cycle. During hospitalization, patients receive chemotherapy and undergo monitoring and management of treatment-related toxicities, including febrile neutropenia (FN) [3, 6]. Given that a substantial proportion of patients with AML fail to achieve long-term remission, strategies that safely reduce hospitalization and maximize time spent at home with family and friends are of considerable importance.

Danish investigators have pioneered outpatient management of AML patients, introducing electronic infusion pumps to administer ICT at home or at day care units, and initiating treatment of FN with intravenous antibiotics (IVAB) on an outpatient basis after an initial evaluation at the hospital [2, 7, 8]. Several studies have evaluated the feasibility of early discharge following induction therapy for AML, reporting reassuring outcomes [7, 9–11]. In these studies, patients with febrile neutropenia were initially hospitalized; however, clinically stable patients could be discharged early and continue treatment with IVAB at home. Other studies evaluating outpatient initiation of treatment for febrile neutropenia have included relatively few patients with AML [12, 13], excluded patients receiving intensive chemotherapy [14, 15], or focused on low-risk populations treated with oral antibiotics [16–18].

The Department of Hematology at Karolinska University Hospital has in close collaboration with Hospital at Home (HaH) in the Stockholm Region in Sweden developed routines for home care of intensively treated patients with AML over the course of more than a decade. At home, patients can receive advanced supportive care including transfusions and parenteral nutrition. During the COVID-19 pandemic in 2020, this collaboration was further expanded to permit clinically stable patients with febrile neutropenia to remain at home while receiving IVAB. This strategy aimed to reduce patients’ risk of SARS-CoV-2 exposure in the hospital emergency department, with the additional benefit of alleviating the critical hospital bed shortage by limiting prolonged inpatient stays.

Outpatient management of FN in intensively treated AML deviates from the current recommendations by the American and European Societies for Medical Oncology [19, 20]. This retrospective study evaluated the safety and feasibility of our novel strategy of at-home management of intensively treated patients with AML.

## Methods

### Aims

The aim of this study was to evaluate safety and feasibility of at-home management of intensively treated AML patients by comparing two Swedish cohorts, with or without access to HaH.

Primary endpoint was a composite endpoint of death or requirement of higher level of care (need for vasopressors, high-flow oxygen/non-invasive ventilation, or admission to the intensive care unit) during the first two treatment cycles (C1 and C2) of ICT for AML. Secondary endpoints included overall survival, days spent in hospital, and outcome of outpatient IVAB treatment of FN.

### Study design and participants

All AML patients diagnosed between January 2020 and October 2023 and treated with intensive induction chemotherapy (ICT) at the Karolinska University Hospital in Stockholm and the Sahlgrenska University Hospital in Gothenburg were included in this retrospective analysis. Since both hospitals are located in major urban centers, it was hypothesized that the patients’ demographics were comparable. All treatment, both in hospital and HaH, was provided free of charge through the Swedish universal healthcare system.

Patients with acute promyelocytic leukemia were excluded.

ICT was defined as standard intensive induction and consolidation in accordance with Swedish national guidelines for AML or clinical trial protocols. C1 is induction; C2 is consolidation if patient achieved remission or re-induction if not. In patients who switched to non-intensive treatment for C2, the primary endpoint was only assessed during C1, when ICT was given.

### Hospital at home in the Stockholm region

**Fig. 1:**
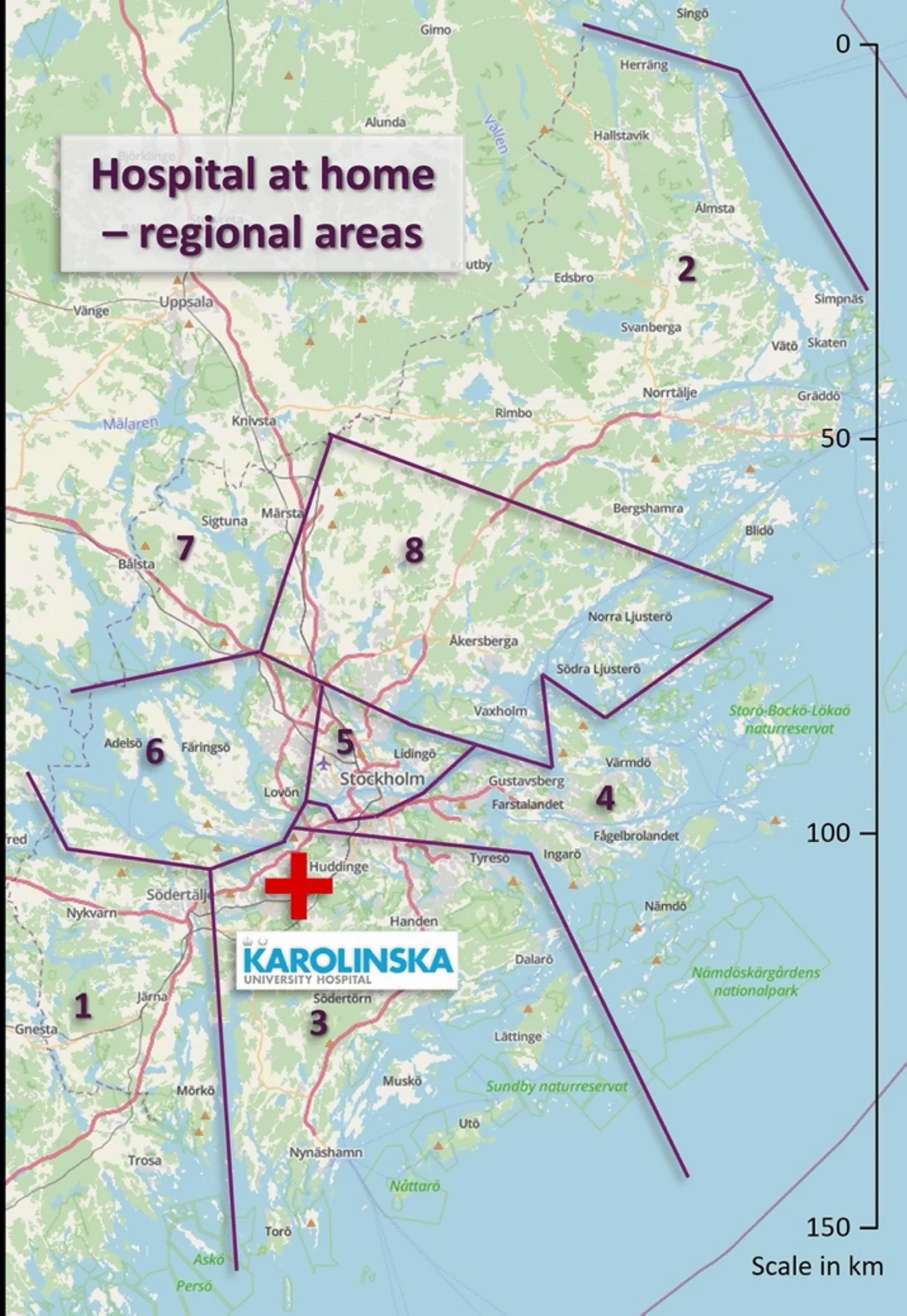
Areas of operation for hospital-at-home units in the Stockholm Region. The Stockholm Region is divided into 8 areas where local Hospital-at-home units provide advanced supportive care. Patients requiring higher level of care are transferred to the Department of Hematology at the Karolinska University Hospital. Some areas include islands in the archipelago, where home care is also provided and helicopter service is available in case of emergencies. Map data: © OpenStreetMap contributors

HaH was provided by 11 healthcare companies, operating from 38 units located in 8 areas in the Stockholm Region (Fig. 1).

HaH provided advanced supportive care at home, including intravenous fluids, parenteral nutrition and medications, and transfusions; transfusions occasionally administered at the local HaH hub. All units had physicians, nurses, and other healthcare professionals with a capacity for telephone support and emergency house calls at all hours. The maximum distance from the patients’ home to the Karolinska University Hospital in Huddinge was around 150 km. The HaH staff received no formal additional training in AML or hematology although they had experience in managing a range of advanced medical conditions within oncology and other fields. During the initial phase of the study, a small number of HaH units were involved, and joint routines were developed in close collaboration. Gradually the program expanded to all HaH units.

HaH teams monitor the patients regularly. Laboratory results are reviewed by both HaH and by hematology ward junior physicians through shared electronic health record system. HaH nurses perform clinical assessments—including evaluation of vital signs, fluid balance, and signs of infection or bleeding—and can initiate urgent treatments, such as IVAB, platelet transfusions or intravenous fluids, in consultation with the HaH physician and the on-call hospital hematologist. A joint management plan for transfusions and neutropenic complications was documented in the shared record system for HaH and the department of hematology. Details of the specific routines for management of AML patients via HaH are described in the supplementary data and in Supplemental Table S1.

### Management and prevention of infections

All patients received prophylaxis with posaconazole, valacyclovir, and ciprofloxacin/levofloxacin during the neutropenic periods, in accordance with national guidelines. G-CSF was routinely given in Stockholm but only to selected patients in Gothenburg. FN was treated according to standard guidelines, i.e., typically piperacillin/tazobactam in stable patients and a carbapenem if more severely ill.

### Data collection

Data were collected via patient electronic health records (TakeCare, CompuGroup GPM in Stockholm; Melior, Oracle Cerner in Gothenburg).

### Ethical considerations

This research complied with the precept of the Declaration of Helsinki. Ethical approval for the retrospective analyses was obtained by the Swedish Ethical Review Authority (Dnr 2024–07172-01). The need for informed consent was waived by the Swedish Ethical Review Authority.

### Statistical analysis

Data were presented as medians with ranges, as appropriate. Nominal variables were compared using chi-square or Fisher’s exact test. Patient survival was estimated using the Kaplan-Meier method and differences between groups were evaluated using the log-rank test. Hazard ratios were estimated using Cox regression. The proportional hazard assumption was verified using Schoenfeld residuals. The Mann-Whitney *U* test was used to compare duration of inpatient care and IVAB. A *p*-value of < 0.05 was considered statistically significant. Statistical analyses were performed using R version 4.4.2 [21].

## Results

### Participants

After exclusion of one patient ineligible for HaH due to residence outside the Stockholm Region, 116 patients were included in Stockholm, of whom 116 and 88 completed C1 and C2, respectively. In Gothenburg, 49 patients were included, with 49 completing C1 and 39 completing C2.

The cohorts were well matched regarding median age, Charlson Comorbidity Index (CCI), and disease risk according to European Leukemia Net (ELN) 2022. The Stockholm cohort had somewhat more patients with severely impaired performance status with Eastern Cooperative Oncology Group performance status (ECOG) 3 or 4 (Table 1).

**Table 1:** Patient and disease characteristics at diagnosis

Characteristic	Stockholm (*N* = 116)	Gothenburg (*N* = 49)
Age (years)—*median (range)*	58 (23–79)	59 (22–76)
Age category—*n (%)*		
20–39	16 (14)	8 (16)
40–59	44 (38)	17 (35)
60–69	30 (26)	13 (27)
70–79	26 (22)	11 (22)
Sex—*n (%)*		
Male *n* (%)	63 (54)	31 (63)
Female *n* (%)	53 (46)	18 (37)
Transformation from MDS or MPN—*n (%)*	5 (4)	2 (4)
Charlson Comorbidity Index—*n (%)*		
2–4	81 (70)	33 (67)
5–6	31 (27)	15 (31)
7 or more	4 (3)	1 (2)
ECOG Performance Status Scale—*n (%)*		
0	28 (24)	12 (24)
1	41 (35)	20 (41)
2	24 (21)	11 (22)
3	17 (15)	6 (12)
4	6 (5)	0 (0)
White blood cell count (10^9^/l)—*median (range)*	11 (1–374)	20 (1–280)
White blood cell count categories (10^9^/l)- *n (%)*		
< 50	77 (66)	36 (73)
50–99	14 (12)	7 (14)
> 100	25 (22)	6 (12)
ELN 2022 Risk Stratification—*n (%)*		
Favorable risk	27 (23)	13 (27)
Intermediate risk	30 (26)	12 (24)
Adverse risk	59 (51)	24 (49)

The first course of ICT was initiated at a higher level of care in four patients, three in Stockholm at the intensive care unit, and one in Gothenburg at the intermediary care unit. Most patients received standard induction chemotherapy with or without a FLT3 inhibitor, in accordance with national guidelines (Table 2).

**Table 2:** Treatment characteristics

**Induction treatment characteristics (C1)**	**Stockholm (*N*** **= 116)**	**Gothenburg (*N*** **= 49)**
*Chemotherapy regimen*—*n (%)*		
DA 3 + 5	85 (73)	35 (71)
DA 3 + 5 and FLT3-inhibitor	28 (24)	13 (27)
FLAG-Ida and FLT3-inhibitor	2 (2)	0 (0)
ACE	1 (1)	1 (2)
*Remission status after cycle 1*—*n (%)*		
CR/CRi or MLFS	97 (84)	34 (70)
> 5% blasts	12 (10)	14 (29)
Unknown	3 (3)	0 (0)
Dead	4 (3)	1 (2)
Febrile episode—*n (%)*	112 (97)	49 (100)
Discharged—*n (%)*	109 (94)	1 (2)
*Re-admissions*—*n (%)*		
No re-admissions	59 (54)	0 (0)
Re-admitted once	46 (40)	1 (2)
Re-admitted twice	4 (3)	0 (0)
Time to readmission (days)—*median (interquartile range)*	5 (6)	1(0)
**Re-induction/consolidation treatment characteristics (C2)**	**Stockholm (*N* = 88)**	**Gothenburg (*N* = 39)**
*Chemotherapy regimen*—*n (%)*		
DA 3 + 5	49 (56)	19 (49)
DA 3 + 5 and FLT3-inhibitor	32 (36)	10 (26)
FLAG-Ida	4 (5)	2 (5)
FLAG-Ida and FLT3-inhibitor	2 (2)	0 (0)
ACE	0 (0)	8 (21)
HiDAC	1 (1)	0 (0)
*Remission status after cycle 2*—*n (%)*		
CR/CRi or MLFS	84 (95)	29 (74)
> 5% blasts	2 (2)	4 (10)
Unknown	1 (1)	5 (13)
Dead	1 (1)	1 (3)
Febrile episode—*n (%)*	71 (81)	36 (92)
Discharged—*n (%)*	88 (100)	39 (100)
*Re-admissions*—*n (%)*		
No re-admissions	38 (43)	10 (26)
Re-admitted once	45 (51)	29 (74)
Re-admitted twice	5 (6)	0 (0)
Time to readmission (days)—*median (interquartile range)*	8 (4)	6 (3)

### Primary outcomes and survival

After C1, 12 of 116 patients (10%) in Stockholm met the primary endpoint (required higher level of care and/or died), compared with 4 of 49 patients (8%) in Gothenburg, *p* = 1.0 (Table 3). Ten of these 12 Stockholm patients were never discharged to HaH but treated as inpatients from the start of treatment until the event occurred, and 5 of them died during C1. Only 2 of the 12 patients were discharged to HaH and later readmitted with need for higher level of care; one received vasopressor treatment for 1 day and one received high-flow oxygen for 1 day (Table 3). No deaths occurred following discharge to HaH.

**Table 3:** Higher level of care death

**Event after induction (C1)**	**Stockholm (*N*** **= 116)**			**Gothenburg (*N*** **= 49)**		
	Never discharged before event	Discharged before event	Total	Never discharged before event	Discharged before event	Total
Any event—*n (%)*	10 (9)	2 (2)	12 (10)	4 (8)	0 (0)	4 (8)
Higher level care—*n (%)*	9 (8)	2 (2)	11 (9)	4 (8)	0 (0)	4 (8)
Death—*n (%)*	5 (4)	0 (0)	5 (4)	1 (2)	0 (0)	1 (2)
**Event after re-induction/consolidation (C2)**	**Stockholm (*N* = 88)**			**Gothenburg (*N* = 39)**		
	Never discharged before event	Discharged before event	Total	Never discharged before event	Discharged before event	Total
Any event—*n (%)*	1 (1)	2 (2)	3 (3)	1 (3)	1 (3)	2 (5)
Higer level care—*n (%)*	1 (1)	2 (2)	3 (3)	1 (3)	0 (0)	1 (3)
Death—*n (%)*	1 (1)	0 (0)	1 (1)	0 (0)	1 (3)	1 (3)

Following C2, 3 of 88 patients (3%) in Stockholm died and/or required higher level of care, compared with 2 of 39 patients (5%) in Gothenburg (*p* = 0.64). In Stockholm, one patient with refractory AML after C1 died in septic shock after C2, without having been discharged, while two patients were discharged with HaH and subsequently readmitted and required higher level of care. In Gothenburg, one patient was found dead at home 10 days after discharge, without having sought medical attention. (Table 3).

We also analyzed the individual components of the primary endpoint. Following C1, death occurred in 5 patients (4%) in the Stockholm cohort and 1 patient (2%) in the Gothenburg cohort (*p* = 1.0). After C2, one patient in each cohort died. Escalation to a higher level of care after C1 was required in 11 patients (9%) in Stockholm and 4 patients (8%) in Gothenburg (*p* = 1.0). Following C2, corresponding rates were 3 patients (3%) and 1 patient (3%), respectively (Table 3).

**Fig. 2:**
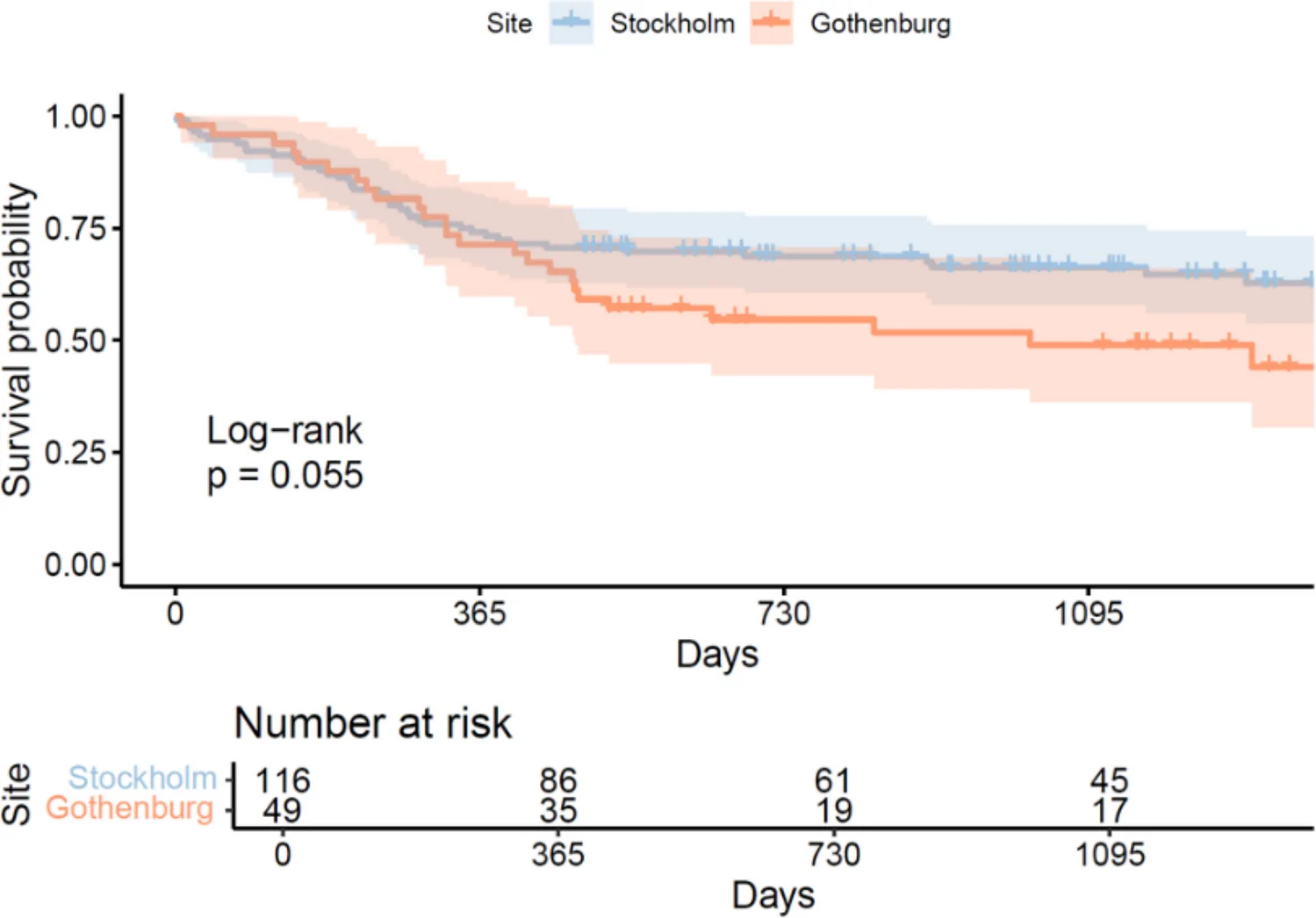
Overall survival. Overall survival of the Stockholm cohort with access to hospital at home and the Gothenburg cohort without access. No difference was observed in 3 and 12 months survival although a trend towards better long-term survival was seen in the Stockholm cohort

The 3- and 12-month survival was comparable in Stockholm and Gothenburg. Beyond the first year, there was a trend toward better overall survival in the Stockholm cohort (Fig. 2). The estimated hazard ratio was lower in Stockholm compared to Gothenburg, but the difference was not statistically significant in neither unadjusted analysis (HR = 0.62, 95% CI 0.38–1.02, *p* = 0.06) nor adjusted for sex, age, CCI, ECOG, and ELN 2022 risk group (HR = 0.65 95% CI 0.38–1.09, *p* = 0.10).

### Duration of inpatient care and readmission rate

**Fig. 3:**
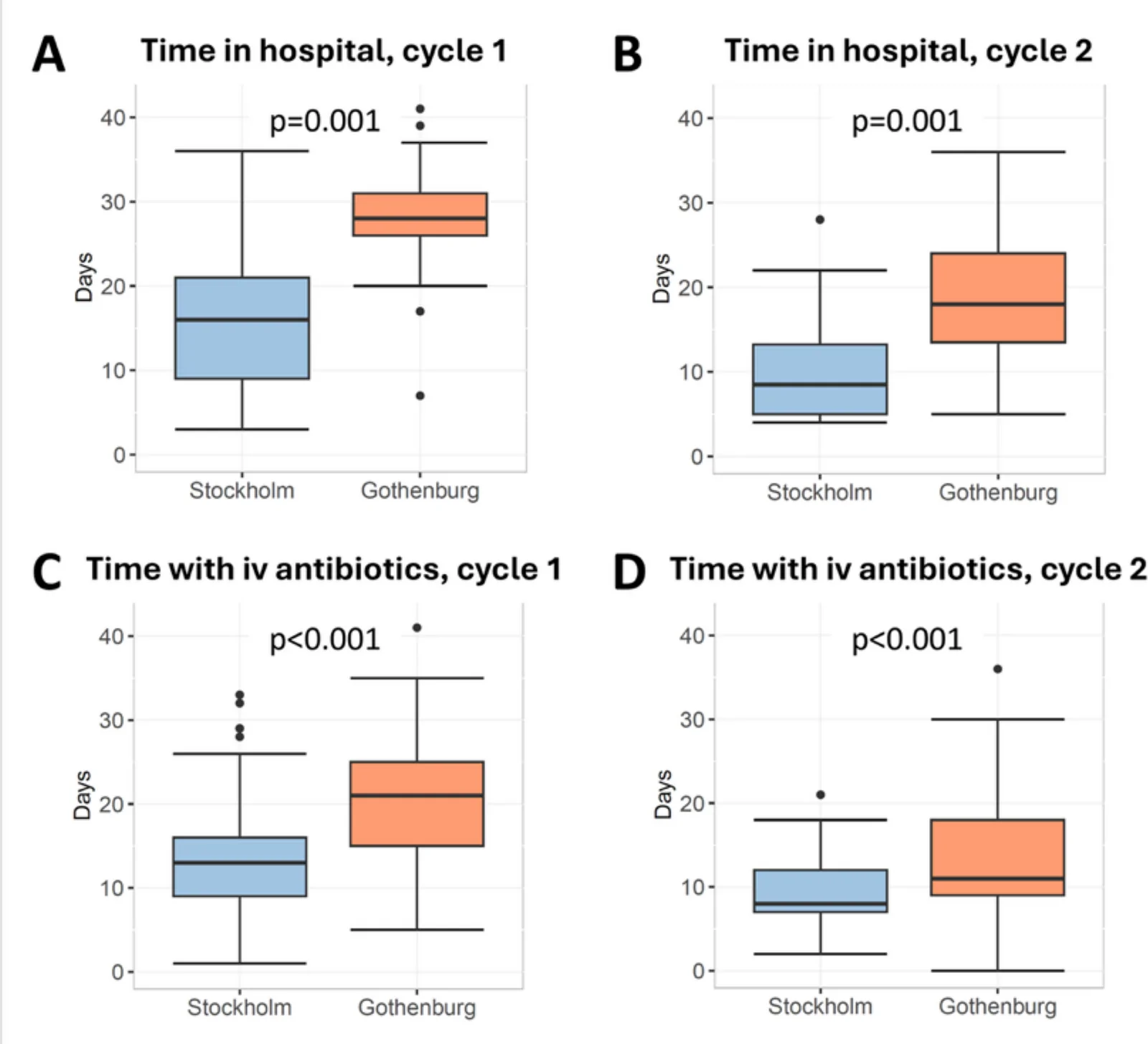
Time in hospital and duration of IV antibiotics. The time spent admitted to hospital was significantly shorter in the Stockholm cohort, with access to hospital at home, both after cycle 1 and 2 (**A**, **B**). The duration with IV antibiotics was significantly shorter in the Stockholm cohort compared with Gothenburg after both cycle 1 and cycle 2 (**C**, **D**)

The median time of inpatient care was shorter in Stockholm compared with Gothenburg after both cycles (C1: 16 vs. 28 days, *p* = 0.001; C2: 9 vs. 18 days, *p* = 0.001; Fig. 3A, B).

For Stockholm patients after C1, being below 65 years of age or having CCI < 5 was associated with a trend of requiring less time in hospital (Supplemental Figure S1).

During cycle 1 (C1), 1 of 49 patients (2%) in the Gothenburg cohort was discharged, compared with 109 of 116 patients (94%) in the Stockholm cohort. In Stockholm, 50 of 109 (46%) discharged patients were readmitted. The reasons for readmission were fever in combination with clinical deterioration (*n* = 11; 22%), other symptoms requiring hospitalization with or without concomitant fever (*n* = 10; 20%), adherence to prior protocol of inpatient FN treatment (*n* = 9, 18%), patient not yet enrolled in HaH (*n* = 9; 18%), persistent fever without clinical deterioration (*n* = 9; 18%), and patient preference (*n* = 2; 4%). After C2, all patients were discharged in both cohorts, although the readmission rate was nominally lower in Stockholm compared with Gothenburg, at 57% and 74%, respectively, *p* = 0.092 (Table 2).

### Duration of intravenous antibiotic therapy and microbiological findings

During C1, 111 (96%) in Stockholm and 48 (100%) in Gothenburg developed FN. The corresponding numbers during C2 were 71 (81%) in Stockholm and 35 (90%) in Gothenburg. After both C1 and C2, the median duration of intravenous antibiotic therapy was shorter in Stockholm compared with Gothenburg (C1, 12 vs. 21 days, *p* < 0.001; C2, 7 vs. 11 days, *p* < 0.001; Fig. 3C, D).

Microbial findings were only available for the Stockholm cohort. The frequency of positive blood cultures was significantly lower among patients who received their entire IVAB treatment at home (11%) compared with those who were admitted (39%) (*p* = 0.0015), likely reflecting the severity of the clinical condition of the patient. The majority of the identified bacteria in the blood were gram-positive (Supplemental Table S2), as expected during quinolone prophylaxis.

### Outpatient administration of intravenous antibiotics in Stockholm

**Fig. 4:**
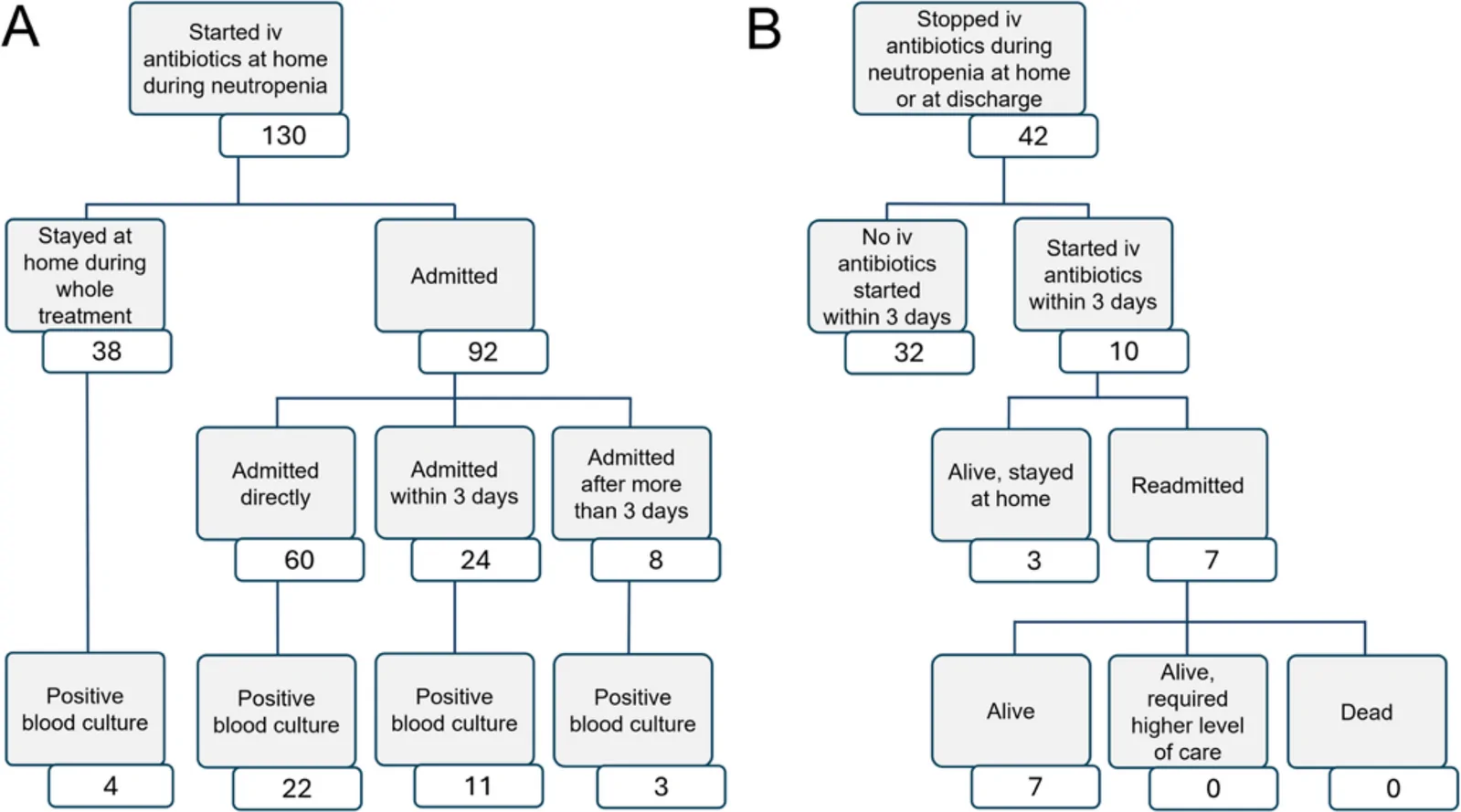
Admittance rate for patients with febrile neutropenia and requirement of reinitiation of IV antibiotics after discontinuation during neutropenia. The Stockholm cohort had access to hospital at home and was evaluated for at-home IV antibiotics in case of febrile neutropenia. Patients treated entirely at home had fewer positive blood cultures. Among admitted patients, over half were admitted directly, and more than a third had positive blood cultures (**A**). After successful treatment, 42 patients discontinued IV antibiotics while still neutropenic; most did not restart antibiotics within 3 days, and none required higher level of care (**B**)

Out of 150 episodes where Stockholm patients were discharged during or prior to neutropenia, FN developed in 130 cases (86.7%). Of these, 38 patients (29%) stayed at home during the entire course of IVAB treatment, whereas 92 (71%) were admitted to hospital. Among admitted patients, the majority (65%) were transferred to hospital directly, after receiving an initial dose of IVAB at home in order to minimize the time from onset of fever to start of IVAB administration (Fig. 4A).

In Stockholm, 15% of all days of IVAB treatment after C1 were given at home compared to 43% after C2. This difference reflected that many patients required IVAB at the time of diagnosis or during the initial course of chemotherapy. After C2, the proportion of days with IVAB administered at home differed depending on age; 54% in patients aged 18–39 years vs. 34% in patients > 60 years.

During the study period, an increasing number of HaH units developed routines for continuous infusion with piperacillin-tazobactam, which facilitated administration at home. This, together with gradually growing experience and optimized routines, resulted in a trend over time that more patients could be managed at home; during 2020–2021, 24% of patients who developed fever after discharge stayed at home during the entire period with IVAB, compared with 34% during 2022–2023 (*p* = 0.29).

### Safety of outpatient discontinuation of intravenous antibiotics during neutropenia

At the time of IVAB discontinuation, most patients had recovered their neutrophil counts. However, in Stockholm, IVAB was stopped in 42 cases during neutropenia, either while being at home or at the time of hospital discharge. Out of these, 32 patients (76%) did not need to restart IVAB again within the next 72 h, while 10 (24%) restarted IVAB again within 72 h, whereof 7 were readmitted to hospital although none required higher level of care or died (Fig. 4B).

### Patients’ experience of home care

There was no structured follow-up of how the patients and their relatives experienced the home treatment via HaH. However, in yearly routine patient questionnaires, nearly all patients were thankful for the opportunity of having access to HaH, including being able to receive transfusions and IVAB at home, as well as emotional support. Many patients also commented on the value of eating their own food and appreciated being in their home environment and spending time with friends and family.

## Discussion

The aim of this retrospective analysis was to assess the feasibility and safety of managing intensively treated AML patients in an outpatient setting using the Stockholm model of HaH. To our knowledge, this approach is unique in that it allows heavily treated and fully neutropenic patients to remain at home for extended periods while receiving advanced supportive care, including transfusions, intravenous antibiotics, and parenteral nutrition, without requiring inpatient assessment unless medically warranted. To evaluate the impact of this novel approach, consecutive AML patients from the two largest Swedish centers, Stockholm with access to HaH, and Gothenburg without access to HaH, were compared.

No differences in probability of short-term complications were observed between the cohorts. Regarding safety, only 4 of 150 episodes (3%) in which patients were discharged during neutropenia resulted in readmission to a higher level of care, a rate comparable to that expected among intensively treated patients with AML developing FN. Additionally, approximately one-third of patients with FN were able to remain at home throughout the entire period with IVAB, a fraction that gradually increased during the study period thanks to optimized routines and growing experience.

Early mortality did not differ between the two cohorts. However, there was a trend toward improved long-term survival in the Stockholm cohort beyond the first year. While this may be due to chance, it is conceivable that increased home-based care has benefits that could support immune function and enhance tolerance to subsequent therapies, including HSCT. Factors such as better nutrition, enhanced mobilization, and overall physical condition may contribute to this.

A crucial factor for managing patients with AML via HaH is a close collaboration with the hematology unit, with joint action plans and standard operating procedures. Logistics are also critical: HaH nurses must be able to perform urgent home visits around the clock and, ideally, administer IVAB as continuous infusions to minimize daily visits.

Outpatient management of FN carries potential risks, especially for patients home alone, including rapid deterioration due to aggressive antibiotic-resistant infections and adverse events such as falls. In case of FN or other severe complications at home, it is of utmost importance that a medically sound decision is made regarding whether to treat the patient at home or to transfer the patient to the hospital. To minimize the risks, the hematology team considers a series of predefined factors as well as the current clinical situation and then makes a joint decision between all parties involved, including the patient and their family. Using this strategy, we demonstrate that patients in Stockholm, with access to HaH, had similar rates of early mortality or severe complications as patients in Gothenburg that were managed without HaH. Also, out of almost 200 episodes of outpatient care of neutropenic AML patients after ICT in Stockholm, only 2% required a higher level of care upon readmission, which is not higher than expected for heavily treated patients in this situation. Early administration of IVAB in patients with FN is associated with reduced morbidity and mortality [22]. The HaH strategy of delivering the IVAB rapidly at home—often within one hour of fever onset—may favorably have influenced the outcome of the infectious episode, even in patients subsequently admitted to hospital.

Patients in Stockholm, managed with HaH, required around half as many days admitted to the hospital after both C1 and C2. During the first two cycles of ICT, the Stockholm patients could spend on average 3 weeks more at home, which amounts to around one third of the entire period. As the experience of home treatment increased and the routines were optimized, a larger proportion of patients were able to stay at home during FN. Also, as expected, younger and more fit patients were also able to stay at home to a greater extent. Several additional factors also likely affected the probability of being treated more at home, such as having a relative present, distance from hospital, compliance, and language skills; however, these were not evaluated in the current study.

The Stockholm patients required 40% fewer days with IVAB compared with the patients in Gothenburg during the period of C1 and C2, which was unexpected. This may reflect differences in local practices, including more liberal use of G-CSF in Stockholm, which can shorten the neutropenic period by up to 5 days [23], and potential differences in prophylactic antibiotic use. Additionally, home-based management in Stockholm may have resulted in faster recovery thanks to greater physical activity and improved nutrition and potentially also a lower risk of acquiring multi-resistant bacteria [24, 25].

In the Stockholm cohort, most patients discontinued IVAB after clinical improvement and neutrophil recovery, a practice generally considered safe. However, neutropenic patients discontinuing IVAB were at the time recommended to be hospitalized for an additional 24–48 h according to the 4th European Conference on Infections in Leukaemia (ECIL-4) guidelines from 2011 [26]. In the Stockholm study cohort, 42 neutropenic patients who had achieved infection control stopped IVAB; over three-quarters did not require to restart IVAB within 3 days, and only 17% were readmitted for recurrent infection, with none needing higher-level care. These data suggest that discontinuing IVAB in selected neutropenic patients is safe, though new infections may occur. The updated ECIL-10 guidelines (2024) [27] now allow close monitoring of neutropenic patients discontinuing IVAB in either inpatient or outpatient settings, thus being in concordance with our clinical practice during the study period.

The patients’ experience of home treatment has largely been very positive. A prospective study addressing the benefits and potential negative consequences of home care in AML, from the patients’ perspective as well as their family members’, including quality of life, depression, nutrition, and loss of muscle mass is currently being performed.

AML treatment is highly resource-intensive, regardless of whether care is delivered in hospital or at home. In Stockholm, the existing HaH infrastructure was utilized alongside AML-specific protocols for transfusions and management of neutropenic complications. Consequently, implementation of home-based AML care did not require additional on-call staffing or other dedicated resources within the HaH service or the hematology department.

Although the present study was not designed to assess the economic impact of home care, access to HaH was associated with a substantial reduction in inpatient hospital days and likely also fewer hospital visits, as transfusions and IVAB could be administered either at home or at local HaH hubs.

Our data suggest that home-based AML care may improve healthcare utilization, provided that a well-established HaH infrastructure is available. As HaH services continue to expand, identifying patient populations that can be managed safely outside the hospital will be essential. Our findings provide practical insights into the implementation and delivery of HaH for patients receiving intensive AML treatment and may inform other healthcare systems considering extending established HaH programs to this patient population.

A major limitation of this study was the relatively small sample sizes which decreased the power to detect rare but potentially severe adverse consequences of home management of FN. A strength of the study was the population-based nature of both cohorts and their similar demographics. AML care in Sweden is coordinated through national guidelines, but minor differences between regions are still present. The most evident in this study was the use of G-CSF in Stockholm, which likely contributed to the shorter periods of IVAB.

Moving forward, the Karolinska University Hospital has expanded at-home care for AML, including offering selected patients consolidation therapy with intensive chemotherapy (ICT) via electronic infusion pumps (since November 2022). To further optimize advanced home care for patients with AML, closer collaboration with selected HaH units would also be valuable. These units could receive more comprehensive training in AML and its related complications, thereby increasing their expertise and facilitating further development of specialized outpatient care, such as at-home monitoring of vital signs, including continuous measurement of body temperature, and the ability to administer additional types of broad-spectrum IVABs as continuous infusions.

To our knowledge, this is the first study evaluating comprehensive outpatient management of FN in AML. Using the Stockholm approach, at-home care with support from HaH appeared safe, associated with significantly more time spent at home, and resulted in fewer days on IVAB. We propose that at-home management of AML offers substantial value to patients both from psychosocial and medical perspectives, with an added bonus of reducing healthcare costs and hospital bed demand.

## Supplementary information

Below is the link to the electronic supplementary material.

Supplementary file1 (PNG 450 kb)

Supplementary file2 (DOCX 156 kb)

Supplementary file3 (PDF 72 kb)

## Data Availability

Aggregated original data can be provided upon request. By Swedish law, we are not at liberty to share individual patient data.
