# Supplementary material for: Hospital at home enables safe and efficient outpatient management of intensively treated acute myeloid leukemia
Source: Support Care Cancer. 2026 Sep 10;34(10):954. doi: 10.1007/s00520-026-11171-2 (PMC13562341; doi:10.1007/s00520-026-11171-2)
Supplement: Supplementary file 2 [file 520_2026_11171_MOESM2_ESM.docx]

### Supplementary data

*Routines for management of AML via hospital at home*

In Stockholm, medically stable AML patients were discharged with HaH after finishing their induction chemotherapy, if deemed appropriate by the attending hematologist. Blood tests were taken at home thrice weekly by a HaH nurse and blood transfusions (both red blood cells and platelets) were administered in accordance with the patient-specific transfusion thresholds. Laboratory values were reviewed by the HaH staff but signed off by junior doctors at the Karolinska University Hospital Hematology ward; both the hospital and HaH utilized the same electronic record system, allowing full access to patient charts as well as laboratory and radiology results. This dual monitoring system enabled HaH physicians to implement appropriate adjustments to supportive care medications, while modifications in oral chemotherapy/targeted drugs or prophylactic therapies were made as needed by physicians at the hematology ward.

The HaH nurses, together with other supporting staff, including the AML contact nurse located at the hospital ward, ensured that the patients have adequate nutritional intake, mobilization, and emotional support. There were no scheduled visits to the hematology department after discharge except for a bone marrow evaluation around day 25, which was followed-up by a telephone call to the patient from the specialist at the hematology ward.

In case of signs of infection or other worrying physical symptoms, the patient contacted the HaH, who dispatched a nurse to visit the patient preferably within 30 minutes, or, if the patient appeared critically ill, called an ambulance directly. The HaH nurse evaluated the patient, including a control of vital signs, and contacted the HaH doctor and hematologist on call to make a joint evaluation of whether to continue home treatment, or to send the patient to the emergency room via ambulance. Decision-making was guided by an overall assessment of the patient’s clinical status and psychosocial factors according; however, no strict criteria were defined for continuation of home-based care (general guide presented in Supplemental Table S1).

All patients with FN had blood cultures performed and IVAB administered. If the patient was deemed suitable for continued home management, IVAB were continued by HaH. As needed, the patient also received intravenous fluid and nutrition. If the nurse, or the patient, at any time judged the condition to deteriorate, the routine was to send the patient to hospital for admission, and to inform the hematologist on call. The patients that remained stable continued IVAB and other supportive care at home.

**Supplemental Table S1.**
Criteria for home-treatment of febrile neutropenia via Hospital-at-Home

1. The patient should preferably have someone present at home at all times;
   in selected cases with mild symptoms a joint decision can be made to home-treat patients living alone

2. The patient should have a stable general condition, be alert and oriented,
   with no signs of hemodynamic instability

3. The patient should be able to reach the hospital within one hour in the event of
   clinical deterioration

| **Supplemental Table S2.** Blood cultures at start of fever at home. | | | | | | |
| --- | --- | --- | --- | --- | --- | --- |
| **Blood culture results** | Treated at home (N = 38) | | Readmitted  (N = 92) | | Total  (N = 130) | |
|  | *Frequency* | *Percent* | *Frequency* | *Percent* | *Frequency* | *Percent* |
| **Negative** | 34 | 89 | 56 | 61 | 90 | 69 |
| **Positive** | 4 | 11 | 36 | 39 | 40 | 31 |
| **Gram-positive** | 3 | 8 | 31* | 34* | 34 | 26 |
| *Strep. mitis* | 1 | 3 | 16 | 17 | 17 | 13 |
| *Coagulase-negative staphylococci* | 2 | 5 | 6 | 7 | 8 | 6 |
| *S. epidermidis* |  |  | 4 | 4 | 4 | 3 |
| *Rothia mucilaginosa* |  |  | 2 | 2 | 2 | 2 |
| *Gemella* |  |  | 1 | 1 | 1 | 1 |
| *Clostridium perfringens* |  |  | 1 | 1 | 1 | 1 |
| *Enterococcus faecium* |  |  | 1 | 1 | 1 | 1 |
| *S. pneumoniae* |  |  | 1 | 1 | 1 | 1 |
| **Gram-negative** | 1 | 3 | 5 | 5 | 6 | 5 |
| *Escherichia coli* |  |  | 3 | 3 | 3 | 2 |
| *Klebsiella pneumoniae* |  |  | 1 | 1 | 1 | 1 |
| *Stenotrophomonas maltophilia* | 1 | 3 |  |  | 1 | 1 |
| *Serratia species* |  |  | 1 | 1 | 1 | 1 |
| * One patient had both Streptococcus mitis and Rothia mucilaginosa in blood cultures. | | | | | | |

**Supplemental Figure S1: Time in hospital stratified by age and comorbidities.**

Explorative subgroup analyses of the time spent in hospital after cycle 1 and 2 were performed by stratifying the patients by age and Charlson Comorbidity Index (CCI). After cycle 1, there was a trend that patients below 65 years or with a CCI below 5 required fewer days in hospital.
